# Supplementary figures and images for: Structural elucidation of the haptoglobin–hemoglobin clearance mechanism by macrophage scavenger receptor CD163
Source: PLoS Biol. 2025 Jul 11;23(7):e3003264. doi: 10.1371/journal.pbio.3003264 (PMC12273918; doi:10.1371/journal.pbio.3003264)

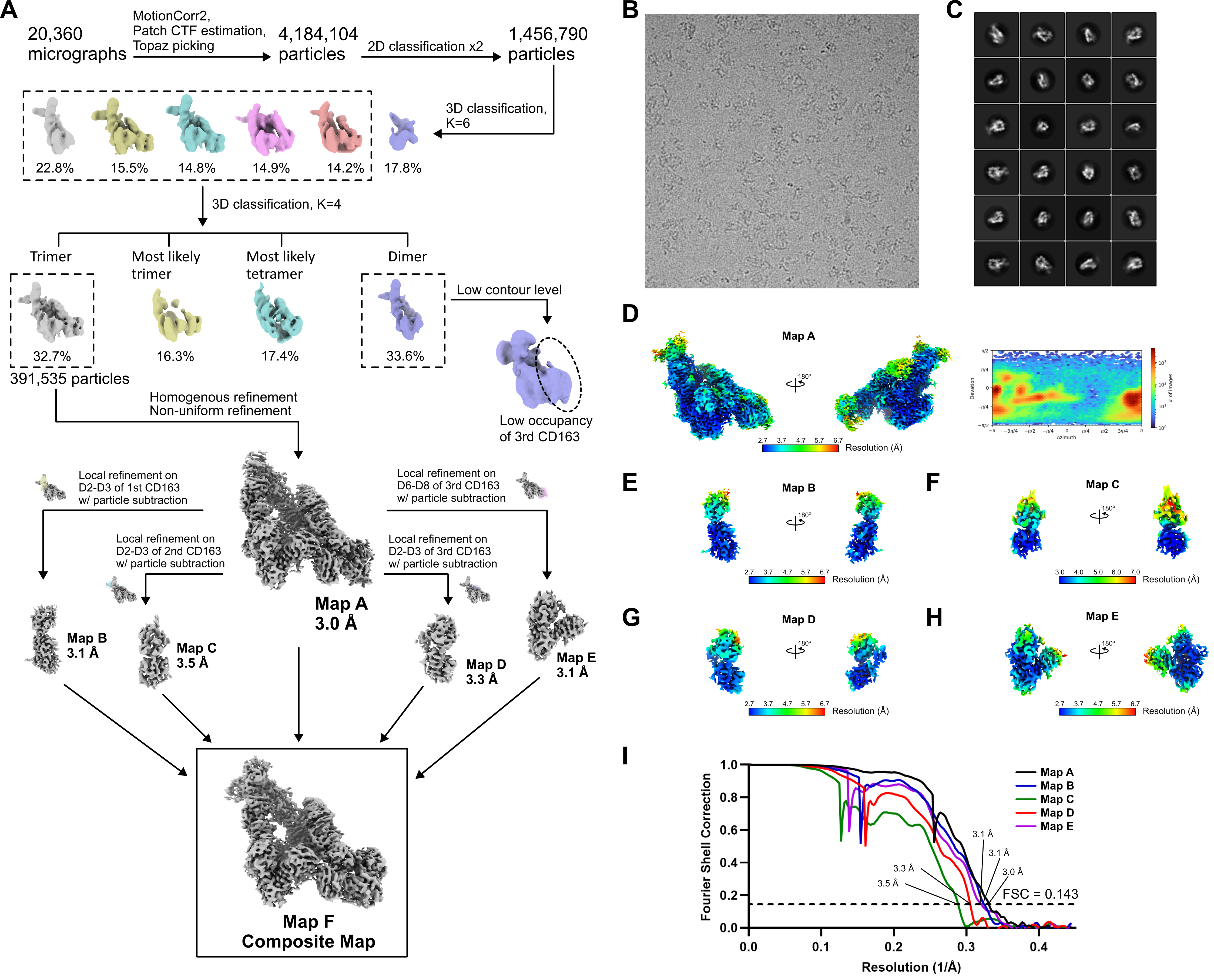

Supplement: S1 Fig — (A) Flow chart of data processing. Details can be found in the Image processing section. (B) Representative cryo-EM micrograph. (C) Representative 2D class averages. (D) Local resolution estimation and orientation distribution plot for the consensus map A. (E–H) Local resolution estimation for the local refinement maps B–E. (I) Gold-standard FSC curves for the consensus maps A and local refinement maps B–E. (TIFF) [file pbio.3003264.s001.tiff]

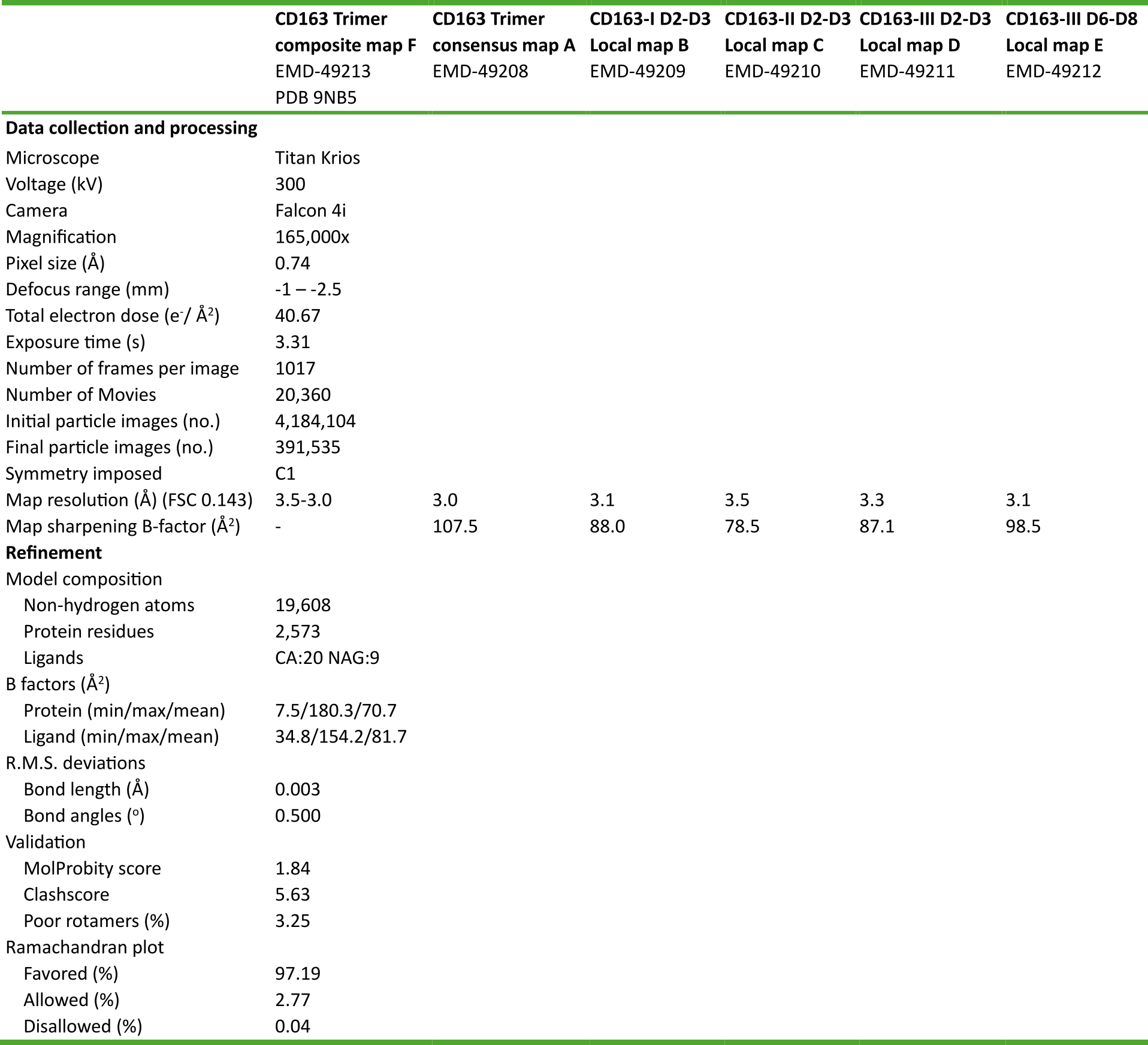

Supplement: S1 Table — (TIFF) [file pbio.3003264.s002.tiff]

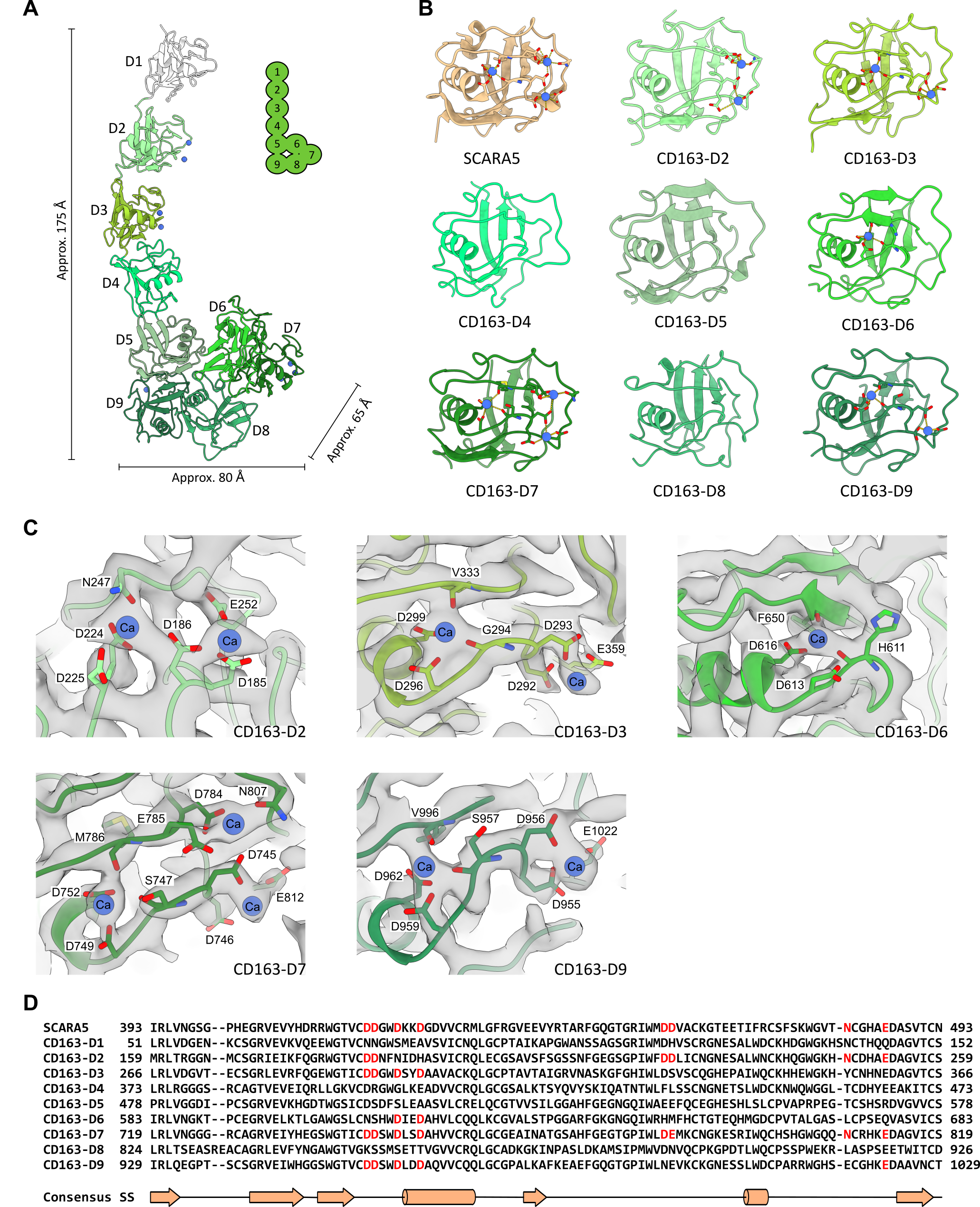

Supplement: S2 Fig — (A) Structure of CD163 ECD. The CD163 ECD structure was assembled by fitting the structure of domain D1, predicted by AlphaFold2, and the structure of domains D2–D9 from the unliganded CD163 model, to the map contoured at a low level. (B) Structures of SRCR domains of SCARA5 (PDB: 7C00) and CD163 (D2 to D9). Residues involved with cation-binding and Ca+2 ions are shown as sticks and spheres, respectively. (C) Ca+2 ions bound to the acidic clusters in CD163 D2, D3, D6, D7, and D9. Residues involving Ca+2 binding and Ca+2 ions bound to these acidic clusters are shown as sticks and spheres, respectively. The cryo-EM map in these regions is shown as gray surface. (D) Multiple sequence alignment of SRCR domains of SCARA5 and CD163 (D1 to D9). Conservation of the cation-binding residues are highlighted in red. (TIFF) [file pbio.3003264.s003.tiff]

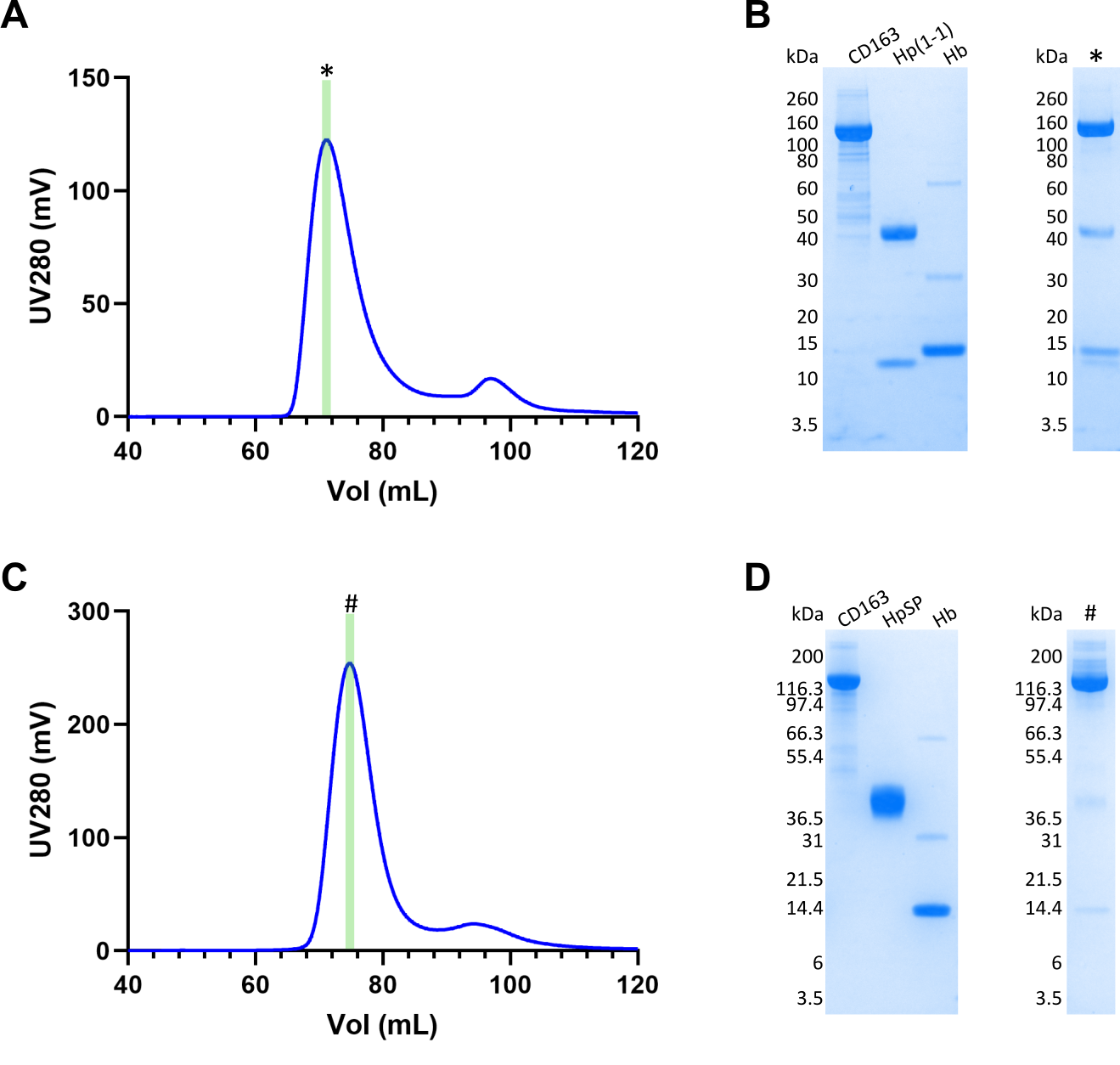

Supplement: S3 Fig — (A) Size-exclusion chromatogram and (B) corresponding SDS–PAGE analysis of the purified CD163/Hp(1–1)Hb complex. The fraction highlighted in the chromatogram was analyzed in the SDS–PAGE reducing gel showing each component of the complex. (C) Size-exclusion chromatogram and (D) corresponding SDS–PAGE analysis of the purified CD163/HpSPHb complex. The fraction highlighted in the chromatogram was analyzed in the SDS–PAGE reducing gel showing the components of the complex. (TIFF) [file pbio.3003264.s004.tiff]

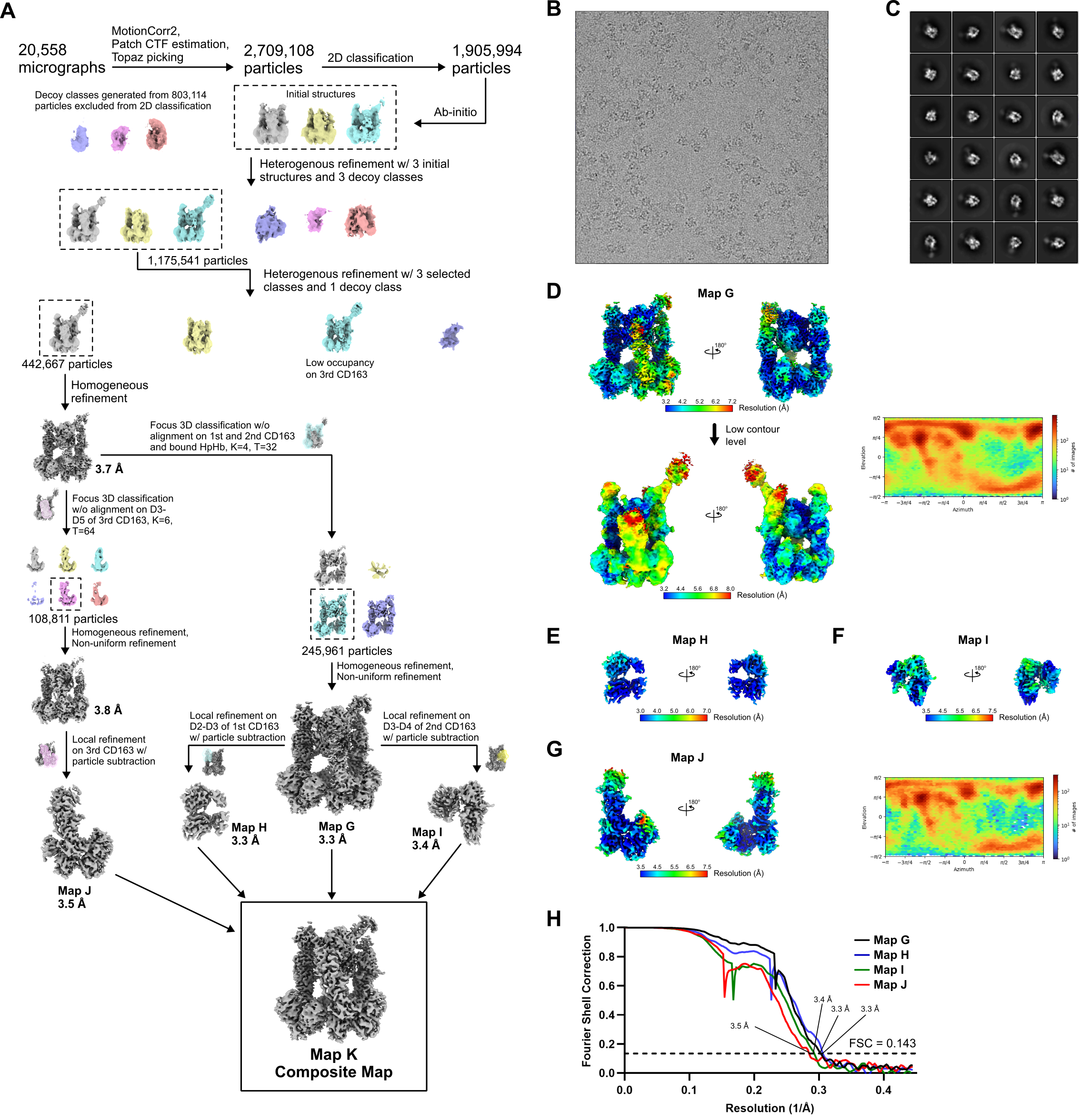

Supplement: S4 Fig — (A) Flow chart of data processing. Details can be found in the Image processing section. (B) Representative cryo-EM micrograph. (C) Representative 2D class averages. (D) Local resolution estimation and orientation distribution plot for the consensus map G. (E–F) Local resolution estimation for the local refinement maps H–I. (G) Local resolution estimation and orientation distribution plot for the local refinement map J. (H) Gold-standard FSC curves for the consensus maps G and local refinement maps H–J. (TIFF) [file pbio.3003264.s005.tiff]

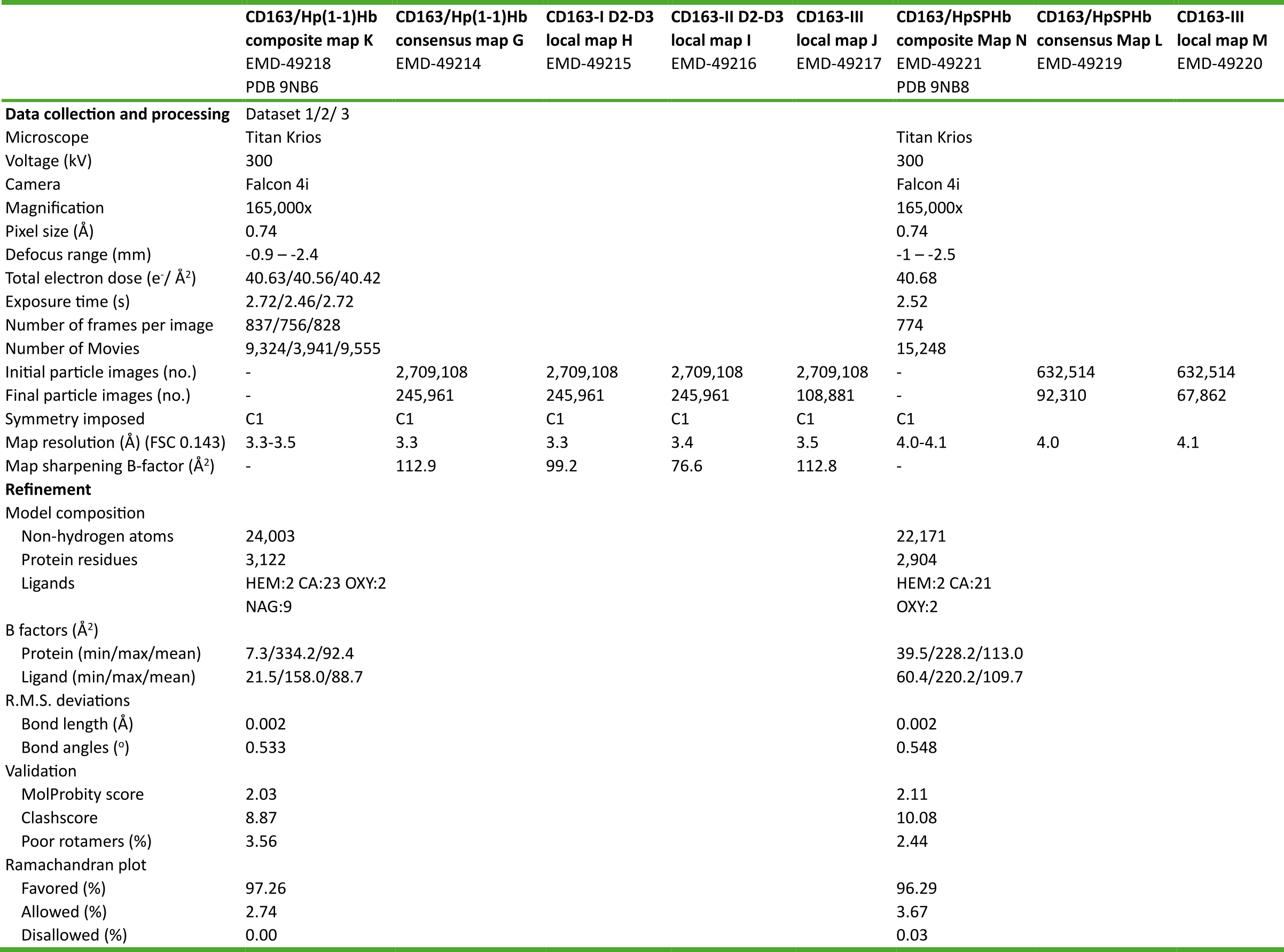

Supplement: S2 Table — (TIFF) [file pbio.3003264.s006.tiff]

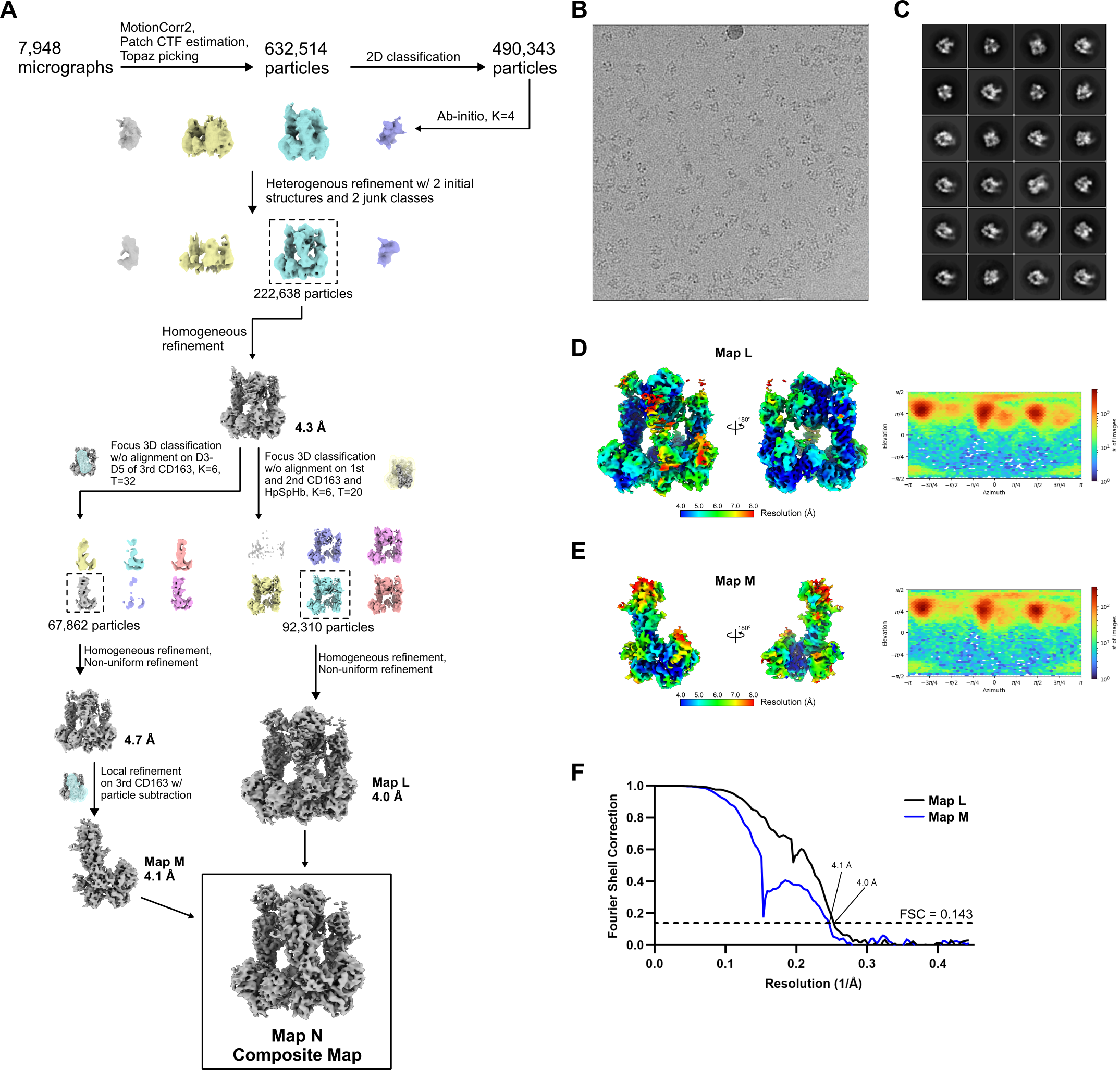

Supplement: S5 Fig — (A) Flow chart of data processing. Details can be found in the Image processing section. (B) Representative cryo-EM micrograph. (C) Representative 2D class averages. (D) Local resolution estimation and orientation distribution plot for the consensus map L. (E) Local resolution estimation and orientation distribution plot for the local refinement map M. (F) Gold-standard FSC curve for the consensus maps L and local refinement maps M. (TIFF) [file pbio.3003264.s007.tiff]

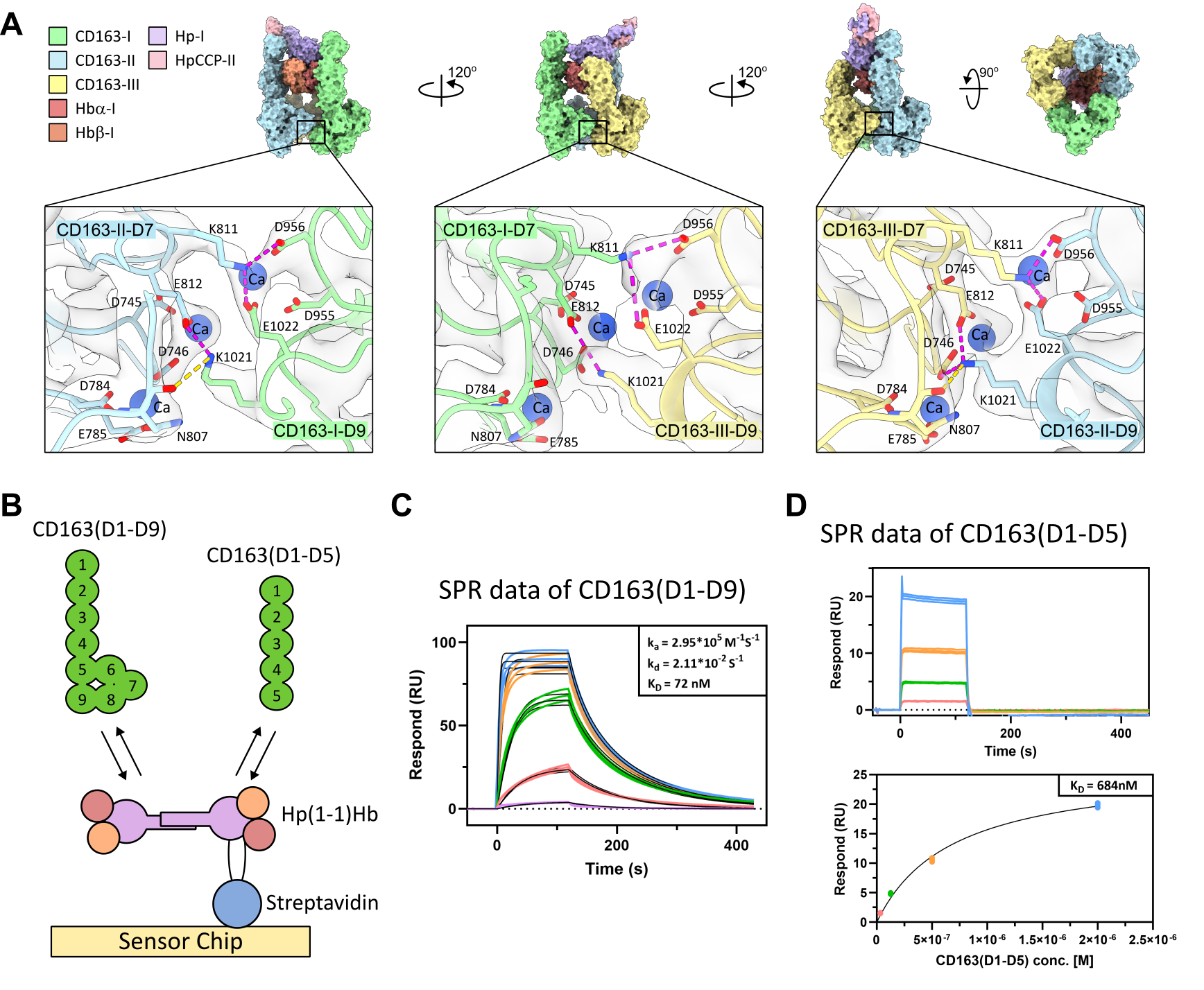

Supplement: S6 Fig — (A) Ca+2-dependent reciprocal electrostatic pairings between D7 and D9 of CD163 subunits. Key residues involved in the interactions and Ca+2 ions bound to the acidic clusters in these interfaces are shown as sticks and spheres, respectively. Magenta dashed lines indicate salt bridges between Lys/Arg and Asp/Glu residues. Yellow dashed lines indicate hydrogen bonds. The cryo-EM map in these regions is shown as gray surface. (B) Schematic illustration of the SPR experiments examining binding of immobilized Hp(1–1)Hb to CD163(D1–D9) or CD163(D1–D5). (C–D) SPR sensorgrams of Hp(1–1)Hb binding to CD163(D1–D9) (C) and to CD163(D1–D5) (D). Each data point is shown (25−2,000 nM, n = 3). The affinity between Hp(1–1)Hb and CD163(D1–D9) was estimated (KD = 72 nM) using kinetic analysis mode. The affinity between Hp(1–1)Hb and CD163(D1–D5) was estimated (KD = 684 nM) using equilibrium analysis mode because the binding was weak and could not be estimated using kinetic analysis mode. Source data for (C–D) can be found in S1 Data. (TIFF) [file pbio.3003264.s008.tiff]

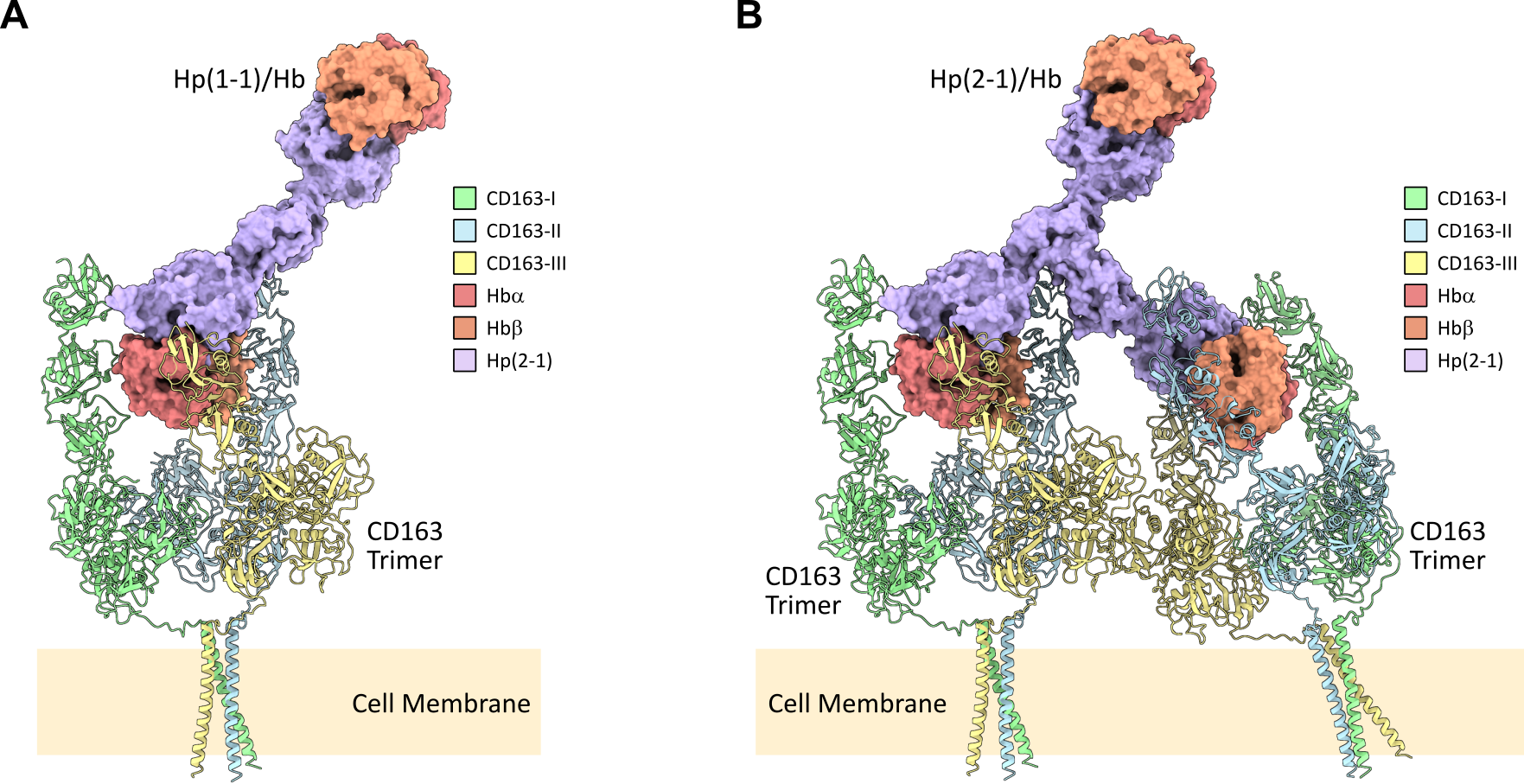

Supplement: S7 Fig — (A) Structural model of a CD163 trimer on the cell-surface bound to Hp(1–1)/Hb. (B) Structural model of two CD163 trimers on the cell surface crosslinked by Hp(2–1)/Hb multimer. All model parts for which the structures are not available – the Hp(2–1)2 complex, the TM helices and the linkers connecting each C-terminus of D9 to the TM domain – were built using the multimer settings in AlphaFold protein structure prediction platform [25]. (TIFF) [file pbio.3003264.s010.tiff]
