# Supplementary material for: Structural elucidation of the haptoglobin–hemoglobin clearance mechanism by macrophage scavenger receptor CD163
Source: PLoS Biol. 2025 Jul 11;23(7):e3003264. doi: 10.1371/journal.pbio.3003264 (PMC12273918; doi:10.1371/journal.pbio.3003264)

**Original and uncropped SDS-PAGE gel image of S4B,**
**Lane 1, 2, 3 and 8 were extracted to create S4B Fig**

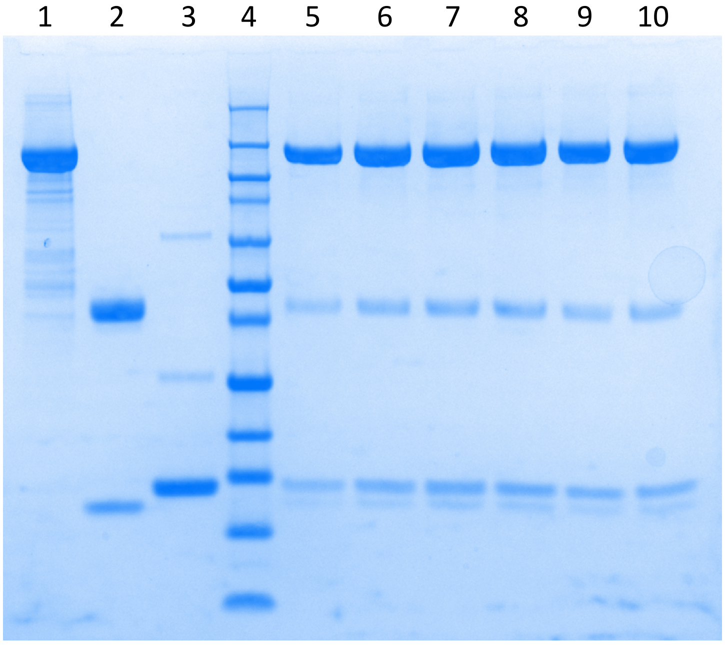


**Original and uncropped SDS-PAGE gel image of S4D,**
**Lane 2, 3, 4 and 6 were extracted to create S4D Fig**

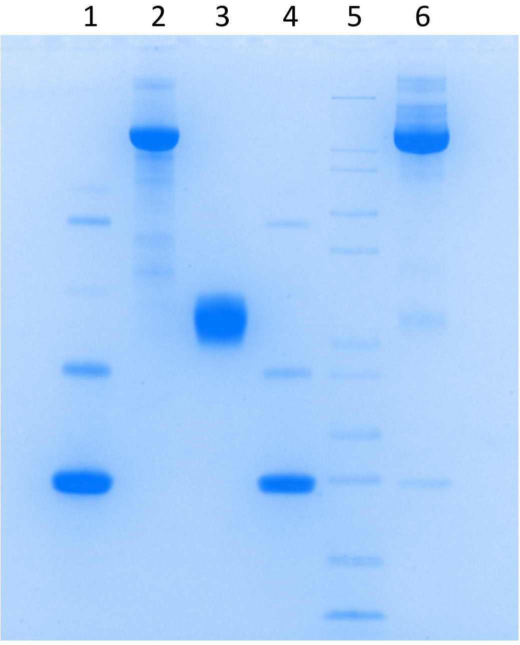

Supplement: S1 Raw Images — (PDF) [file pbio.3003264.s012.pdf]
