# Supplementary material for: Structural elucidation of the haptoglobin–hemoglobin clearance mechanism by macrophage scavenger receptor CD163
Source: PLoS Biol. 2025 Jul 11;23(7):e3003264. doi: 10.1371/journal.pbio.3003264 (PMC12273918; doi:10.1371/journal.pbio.3003264)
Supplement: Attachment — Submitted filename: CD163HpHb_point-by-point-response_20250527.pdf [file pbio.3003264.s015.pdf]

Point-by-point response

Reviewer #1:

The scavenger receptor CD163 plays a critical role in cellular defense by mediating macrophage internalization of free hemoglobin (Hb) during intravascular hemolysis, thereby mitigating vascular and renal tissue damage. In this study, the authors provide critical structural and mechanistic insights into CD163's cargo recognition process by resolving its ligand-free (3.0 Å) and ligand-bound (3.3 Å) conformations. Key findings include the identification of an asymmetric, autoinhibitory trimeric state of unliganded CD163, which sterically blocks the binding site for the haptoglobin-hemoglobin (HpHb) heterodimer. Upon HpHb binding, the receptor transitions to a near-symmetrical 3:1 CD163/HpHb complex. The authors further demonstrate that calcium ions, acting at subunit interfaces, are indispensable for stabilizing CD163 self-assembly, as EDTA-mediated Ca^{2+} chelation disrupts trimerization and reverts the receptor to a monomeric state. This calcium-dependent network is proposed to regulate conformational switching between ligand-free and ligand-bound states, offering a compelling mechanistic framework for the spatiotemporal control of HpHb clearance.

The manuscript is well-structured, methodologically rigorous, and addresses a biologically important question. The conclusions are supported by the data, and the comparative analysis with previous structural work strengthens the novelty of the findings. This study is suitable for publication in PLOS Biology following minor revisions to address the following points:

We very much appreciate the referee's comments and thank him or her for the very constructive feedback.

- The role of Ca2+ in the CD163/HpHb complex assembly is demonstrated through the bulk removal of Ca2+ by EDTA. However, the authors already showed that EDTA treatment could dissemble the unliganded CD163 trimer into monomers. Structure-guided functional-separation mutants would strengthen the mechanistic claims and rule out indirect EDTA effects.

We agree that engineering structure-guided mutant variants of CD163 would be very interesting and highly valuable. Unfortunately, due to recent departmental restructuring, our protein structure support for this project has been discontinued, preventing us from moving

forward with this work. While we are unable to continue this work, several studies have identified and reported mutations in CD163 that impair calcium ion or HpHb binding, supporting our structural findings and offering valuable insights into its functional mechanisms while ruling out indirect EDTA effects.

For example, in our description of the Ca^{+2}-mediated electrostatic network within the CD163/HpHb complex (lines 178-180), we reference the mutagenesis data published by Nielsen et al. (JBC 2013), which demonstrated that mutating Asp or Glu residues within the acidic cluster responsible for Ca^{+2} binding abolished CD163-HpHb interaction. Likewise, Etzerodt et al. (Nature Communication 2024) found that mutating Asp residues within the acidic cluster responsible for Ca^{+2} binding at the CD163-CD163 interface (between D7 and D9 of adjacent subunits) disrupted receptor trimerization. These mutagenesis studies align with our structural findings, emphasizing the critical role of this acidic cluster in establishing reciprocal hydrogen bonds and salt bridges essential for CD163 trimerization at the base. Their work is cited in the discussion section (lines 351–353).

- Panels B-D in Fig.4 are visually overcrowded. The authors may simplify the presentation by highlighting a single subunit with sharper color contrast.

In response to the reviewer's suggestion, we modified the color of each CD163 subunit in Fig 4 and simplified the presentation in Fig 4B-4D. We are grateful to the referee for pointing this out.

- "Hp(1-1)Hp" should be "Hp(1-1)Hb" in Fig.4. legend.

We thank the reviewer for catching this typo. We corrected the typo in the revised version of the manuscript.

Reviewer #2:

The study by Huang et al. reports the structure and mechanism of CD163's binding and import of the haptoglobin-hemoglobin complex. CD163 is a type 1 membrane protein responsible for binding and clearing the circulating HpHb complex. This work resolves the CryoEM structure of CD163 alone and bound to the HpHb complex. These results reveal a significant structural change between the bound and apo states, where the binding site is sequestered in the absence of substrate. These structural changes appear highly dependent upon structural calcium ions at the CD163:CD163 and CD163:HpHb interfaces.

The study is carefully designed, thorough, and well carried out. Generally, the results are properly analyzed and the scientific conclusions are clearly presented and well justified. There is no question the paper should be published. However, I have a few modest points regarding interpretation of their data and the mechanistic model.

We very much appreciate the referee's positive comments and thank him or her for the very constructive feedback.

Major concerns:

- The authors have clearly resolved the CD163/HpSPHb complex from their CryoEM dataset. However, the corresponding gel for this sample (S4D Fig) appears to show only sub-stoichometric amounts HpSP and Hb in the peak fraction, relative to CD163. How do the authors explain this discrepancy?

This question can be addressed by considering the structural compositions of Hp(1-1)Hb and HpSPHb. Hp(1-1)Hb forms a dimeric complex consisting of two Hp1/Hbα/Hbβ protomers linked by a disulfide bond and beta-strand swap within the CCP domain of Hp1. In contrast, HpSPHb is a monomer composed of one HpSP, one Hbα, and one Hbβ due to the deletion of the CCP domain. Despite their distinct structural configurations, both dimeric Hp(1-1)Hb and monomeric HpSPHb assemble into complexes with three CD163 subunits. Consequently, Hp(1-1)Hb complexes contain two copies of Hp1, Hbα, and Hbβ in one CD163/Hp(1-1)Hb complex, whereas HpSPHb complexes have only one copy of HpSP, Hbα, and Hbβ in one CD163/HpSPHb complex.

The observed discrepancy in gel density can be explained by comparing the CD163/HpSPHb sample (S4D Fig) with the CD163/Hp(1-1)Hb sample (S4B Fig) used for cryo-EM. Although the bands for CD163 show similar abundance in both reducing SDS-PAGE gels, the bands corresponding to HpSP, Hbα, Hbβ appear weaker in the CD163/HpSPHb gel, reflecting the

presence of only one copy of these components, compared to the two copies present in the CD163/Hp(1-1)Hb complex.

- Similarly in S4B Fig, there appears to be two bands in the Hp1-1 samples, and both are carried over into the complex peak. Is this additional component identified in the sample and/or apparent in the CryoEM dataset?

This question can also be examined in light of the following considerations. Hp(1-1) consists of two Hp1 chains, originally synthesized as a single polypeptide before undergoing cleavage into chains α and β in the endoplasmic reticulum. The α chain, primarily covering the CCP domain, and the β chain, covering the SP domain, are linked via a disulfide bond. Consequently, Hp(1-1) samples appear as two bands in a reducing SDS-PAGE gel. Both Hp1 α and β chains were accurately mapped in the complex structure using the cryo-EM data.

- The clash score in the CD163/HpSPHb structure appears excessively high, particularly in light of the modest resolution. This should be re-refined with optimized restraints.

We appreciate the referee for highlighting this important point. Following the reviewer's recommendation, we re-refined the structure of CD163/HpSPHb using optimized restraints, which lowered the clash score from 17.07 to 10.08, making it comparable to that for the CD163/Hp(1-1)Hb structure (8.87).

- How the Ca+2 ions were identified is not explained. A justification for modeling these unknown densities as calcium, relative to other possible ions, should be included.

The identification of Ca^{+2} ions was guided by the following criteria: (1) distinct, well-defined density peaks in cryo-EM maps, surpassing a contour level of 8 sigma; (2) the local amino acid environment and coordination chemistry, primarily involving negatively charged Asp and Glu clusters, indicating their interaction with positively charged cations; and (3) the presence of Ca^{+2} ions at 2–5 mM $CaCl_2$ in the protein buffer, consistent with the Ca^{+2}-binding properties of SRCR domains. Following reviewer's suggestion, we added a paragraph detailing these criteria in the Material and Method section (line 487-491).

- The mechanism for HpHb binding by CD163 is not entirely clear from the author's model and results. If several calcium ions stabilize both the auto-inhibited and bound

state, how does this transition occur? Do they need to be released and then re-bind in the conformational change, and does this have consequences for the calcium-dependence of this structural transition.

We appreciate the referee bringing up this question, which is both nuanced and thought-provoking. Given the absence of direct observational evidence, we propose the following considerations based on our biochemical data and structural insights.

Both the ligand-free and ligand-bound structures of CD163 share common Ca^{+2}-binding sites, indicating that Ca^{+2} is integral to the trimeric CD163 architecture in both states. The consistent presence of calcium ions in autoinhibitory and ligand-bound configurations ensures the ability of the receptor to dynamically shift between these states upon ligand binding. We suggest that this conformational shift represents a calcium-bound transition rather than a cycle of calcium release and rebinding. In this process, ligand engagement triggers a substantial structural rearrangement of CD163, rendering preoccupied calcium binding sites accessible to the natural ligand, while simultaneously amplifying and expanding calcium-driven interactions between receptor subunits at the base. Ligand binding and Ca^{+2} work in concert to achieve complete engagement, with the transition between states involving a dynamic competition between the natural ligand and the autoinhibited conformation of the receptor.

We also suggest that ion-mediated electrostatic interactions, rather than an extensive shape-based fit, are key to driving this transition, providing structural flexibility and rotational freedom. This adaptability supports dynamic conformational changes in receptor subunits during HpHb binding, while also working alongside pH to regulate ligand release in low calcium and lower pH conditions in the endosomal compartments.

- Figure 4 panels B, C, and D, the colors are far too pale and therefore not visible on a screen or printed. These should be revised

In response to the reviewer's suggestion, we modified the color of each CD163 subunit in Fig 4 and simplified the presentation in Fig 4B-4D.

Minor concerns:

- The color for salt bridges in Figure 3 is too close to the colors for protein and density and therefore difficult to see. We suggest changing this.

In response to the reviewer's suggestion, we changed the salt bridge color to magenta in Fig 1 and Fig 3.

Trivial concerns:

- The authors occasionally mix single-letter and three-letter amino acid codes, even within the same sentence (such as lines 191-193). This is particularly confusing as the CD163 domains are also described using a single-letter and number code. Therefore, a three-letter code should be used throughout for clarity.

Thank you for pointing this out. We carefully reviewed the manuscript to ensure that all mentioned amino acid residues now appear as three-letter codes in the revised version.
